# Successful underwater endoscopic submucosal dissection for ulcerative colitis-associated neoplasia using a synchronized irrigation fluid management system

**DOI:** 10.1055/a-2658-0148

**Published:** 2025-08-01

**Authors:** Mayo Tanabe, Takemasa Hayashi, Daijiro Shiomi, Kuniko Iihara, Naoyuki Uragami, Noboru Yokoyama, Haruhiro Inoue

**Affiliations:** 1 Digestive Diseases Center, Showa Medical University Koto Toyosu Hospital, Koto-ku, Japan; 2 Digestive Diseases Center, Showa Medical University Northern Yokohama Hospital, Yokohama, Japan; 3 Department of Pathology, Showa Medical University Koto Toyosu Hospital, Koto-ku, Japan

Underwater endoscopic submucosal dissection (UESD) performed with the water-pressure method (WPM) facilitates submucosal exposure and lesion manipulation 1 2 . Additionally, the integration of synchronized water irrigation with the electrosurgical unit using the Erbe Irrigation Pump 2 (EIP2; Erbe Elektromedizin GmbH, Tübingen, Germany) system has been reported 3 , further refining this approach by overcoming visualization challenges and improving procedural efficiency. This video case report illustrates the first clinical application of this innovative technique in treating ulcerative colitis-associated neoplasia (UCAN).

A 66-year-old man with a 10-year history of ulcerative colitis underwent surveillance colonoscopy, which revealed a flat 0-IIb lesion in the sigmoid colon ( Fig. 1 ). Biopsy confirmed tubular adenocarcinoma consistent with UCAN. Although total proctocolectomy is generally recommended for lesions beyond high grade dysplasia, the patient strongly preferred organ-preserving endoscopic resection.

**Fig. 1:**
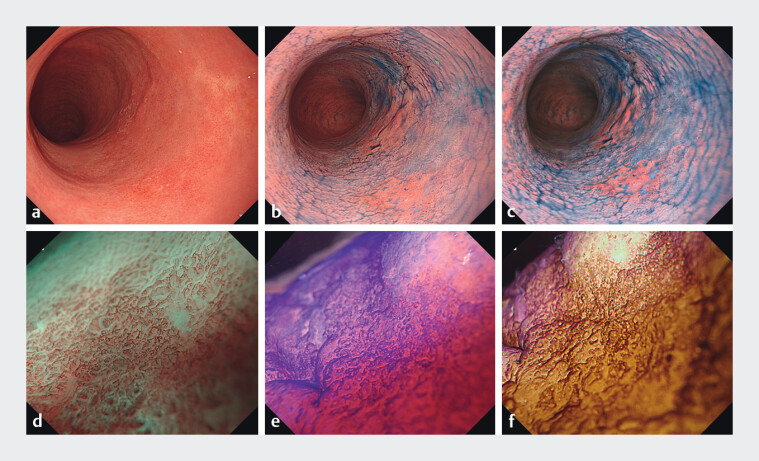
Preoperative endoscopic image of ulcerative colitis-associated neoplasia (Olympus GF-1200Z, EVIS-X1; Olympus, Tokyo, Japan). **a** White-light imaging showing two discontinuous, flat, reddish areas with adjacent scarring. **b** Chromoendoscopy with indigo carmine. **c** Texture and color enhancement imaging after indigo carmine. **d** Magnified narrow-band imaging displaying an irregular surface and vascular pattern with a distinct demarcation line. **e** Crystal-violet staining demonstrating a Kudo type VI-low pit pattern. **f** Red dichromatic imaging emphasizing pit irregularity.

UESD with WPM was performed under complete saline immersion. Continuous hydrostatic pressure provided durable mucosal elevation, mitigated submucosal fibrosis, and enhanced visualization of the fat-rich submucosa ( Fig. 2 , Video 1 ). The EIP2 irrigation system was connected to the endoscope’s waterjet channel and synchronized with the VIO 3 electrosurgical generator (Erbe Elektromedizin GmbH) ( Fig. 3 ). Electrosurgical cutting automatically triggered pressurized irrigation, eliminating bubbles and maintaining a consistently clear visual field. En bloc resection was achieved without muscular injury. Histopathology showed well-differentiated adenocarcinoma, pT1b (submucosal depth 1100 µm), with tumor budding grade 2, infiltration pattern β, no lymphatic or vascular invasion, and negative horizontal and vertical margins for a R0 resection. The postoperative course was uneventful, and the patient was discharged on Day 4.

**Fig. 2:**
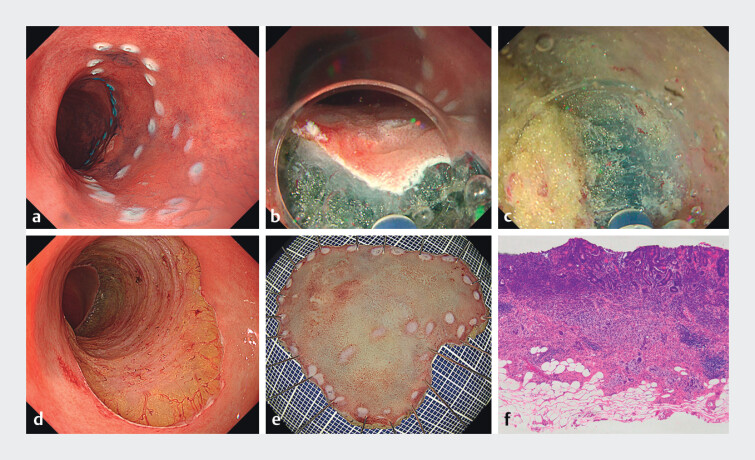
Intraoperative and histological findings of underwater endoscopic submucosal dissection with water-pressure method. **a** Circumferential marking. **b** Incision through thickened muscularis mucosa to access the submucosal layer. **c** Despite the presence of fat-rich submucosa, a clear visual field was maintained under saline immersion, with no bubble interference. **d** Ulcer base after resection with intact muscularis propria. **e** Macroscopic specimen. **f** Histology confirming pT1b adenocarcinoma with negative horizontal and vertical margins.

**Fig. 3:**
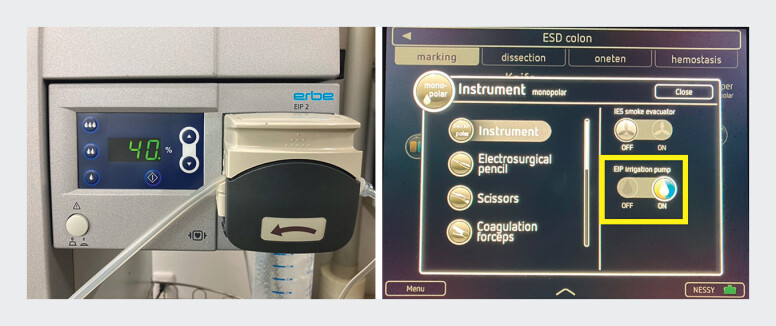
The Erbe Irrigation Pump 2 water irrigation system and the VIO 3 electrosurgical unit (Erbe Elektromedizin GmbH, Tübingen, Germany).

Underwater endoscopic submucosal dissection using the Erbe Irrigation Pump 2 (Erbe Elektromedizin GmbH, Tübingen, Germany) irrigation system for ulcerative colitis-associated neoplasia. The synchronized automatic irrigation enabled bubble-free visualization and stable submucosal dissection, allowing safe and effective resection of fibrotic, fat-rich lesions.Video 1

This case represents the first reported use of UESD with WPM for UCAN, demonstrating its feasibility for lesions with fibrosis and fat-rich submucosa, and allowing for smooth and safe dissection. The integration of the EIP2 system enabled a bubble-free operative field, further enhancing the efficacy and safety of the procedure. Further studies are needed to evaluate its broader applicability in similar clinical scenarios.

Endoscopy_UCTN_Code_TTT_1AQ_2AD_3AD

## Notes

Endoscopy E-Videos https://eref.thieme.de/e-videos
*E-Videos* is an open access online section of the journal *Endoscopy* , reporting on interesting cases and new techniques in gastroenterological endoscopy. All papers include a high-quality video and are published with a Creative Commons CC-BY license. Endoscopy E-Videos qualify for HINARI discounts and waivers and eligibility is automatically checked during the submission process. We grant 100% waivers to articles whose corresponding authors are based in Group A countries and 50% waivers to those who are based in Group B countries as classified by Research4Life (see: https://www.research4life.org/access/eligibility/ ). This section has its own submission website at https://mc.manuscriptcentral.com/e-videos .
